# Digital Micromirror Device (DMD)-Based High-Cycle Torsional Fatigue Testing Micromachine for 1D Nanomaterials

**DOI:** 10.3390/mi7030049

**Published:** 2016-03-14

**Authors:** Chenchen Jiang, Dayong Hu, Yang Lu

**Affiliations:** 1 Department of Mechanical and Biomedical Engineering, City University of Hong Kong, Kowloon, Hong Kong, China; ccjiang3-c@my.cityu.edu.hk (C.J.); hudayong@buaa.edu.cn (D.H.); 2 Center for Advanced Structural Materials (CASM), Shenzhen Research Institute, City University of Hong Kong, Shenzhen 518057, China; 3 Department of Aircraft Airworthiness Engineering, School of Transportation Science and Engineering, Beihang University, Beijing 100191, China

**Keywords:** microelectromechanical systems (MEMS), nanomechanics, nano-fatigue, digital micromirror device (DMD), torsion, nanowire

## Abstract

Fatigue behavior of nanomaterials could ultimately limit their applications in variable nano-devices and flexible nanoelectronics. However, very few existing nanoscale mechanical testing instruments were designed for dedicated fatigue experiments, especially for the challenging torsional cyclic loading. In this work, a novel high-cycle torsion straining micromachine, based on the digital micromirror device (DMD), has been developed for the torsional fatigue study on various one-dimensional (1D) nanostructures, such as metallic and semiconductor nanowires. Due to the small footprint of the DMD chip itself and its cable-remote controlling mechanisms, it can be further used for the desired *in situ* testing under high-resolution optical or electron microscopes (e.g., scanning electron microscope (SEM)), which allows real-time monitoring of the fatigue testing status and construction of useful structure-property relationships for the nanomaterials. We have then demonstrated its applications for testing nanowire samples with diameters about 100 nm and 500 nm, up to 1000 nm, and some of them experienced over hundreds of thousands of loading cycles before fatigue failure. Due to the commercial availability of the DMD and millions of micromirrors available on a single chip, this platform could offer a low-cost and high-throughput nanomechanical solution for the uncovered torsional fatigue behavior of various 1D nanostructures.

## 1. Introduction

Due to their importance as building blocks for nanoscale electronics and electro-mechanical systems in numerous advanced engineering applications, such as flexible electronics/wearable electronics, bio-integrated electronics [1,2,3,4], one-dimensional (1D) nanoscale materials and structures, such as nanowires and nanotubes, have been extensively investigated in the past decades [5,6,7]. However, the ability to achieve the full potential of aforementioned new technologies in these fascinating applications is ultimately limited by the mechanical reliability of these one-dimensional building blocks [8,9,10,11,12]. In particular, fatigue, or the progressive and localized structural damage that occurs when a material is subjected to cyclic loading, is a commonly encountered mode of failure in nanomaterial components, which ultimately determines their device stability and lifetime. Despite substantial research that has been recently devoted to the fatigue behavior of individual 1D nanostructures subjected to cyclic tensile or bending loading [13,14,15,16,17,18], torsional fatigue testing remains little reported, largely due to the challenges of performing high cyclic torsional loading on such exceedingly small samples. Therefore, designing and developing of appropriate testing platforms are the greatest challenges in the current nanomechanics community.

Recently, microelectromechanical systems (MEMS)-based testing platforms have emerged as powerful and versatile tools to interrogate low-dimensional micro- and nanostructures, and a number of MEMS devices have been successfully developed to investigate the mechanical strength and plastic deformation for various kinds of 1D nanomaterials [19,20,21]. However, the dynamic characterization for important fatigue behavior, especially torsional fatigue, remains highly challenging, mainly due to the inherent limitations of existing MEMS designs, such as the loading frequency is not high enough for prolonged fatigue experiments, and the out-plane movement is not available for torsional straining. Here, instead of designing a dedicated MEMS device, we adopted a commercially available MEMS product DMD (Digital Micromirror Device, by Texas Instruments, Dallas, TX, USA) for the desired cyclic torsional loading for individual nanowires [22,23,24], and used this innovative platform to study the intriguing high-cycle fatigue behavior for a few representative nanowire samples.

## 2. Platform Development and Methodology

**Figure 1:**
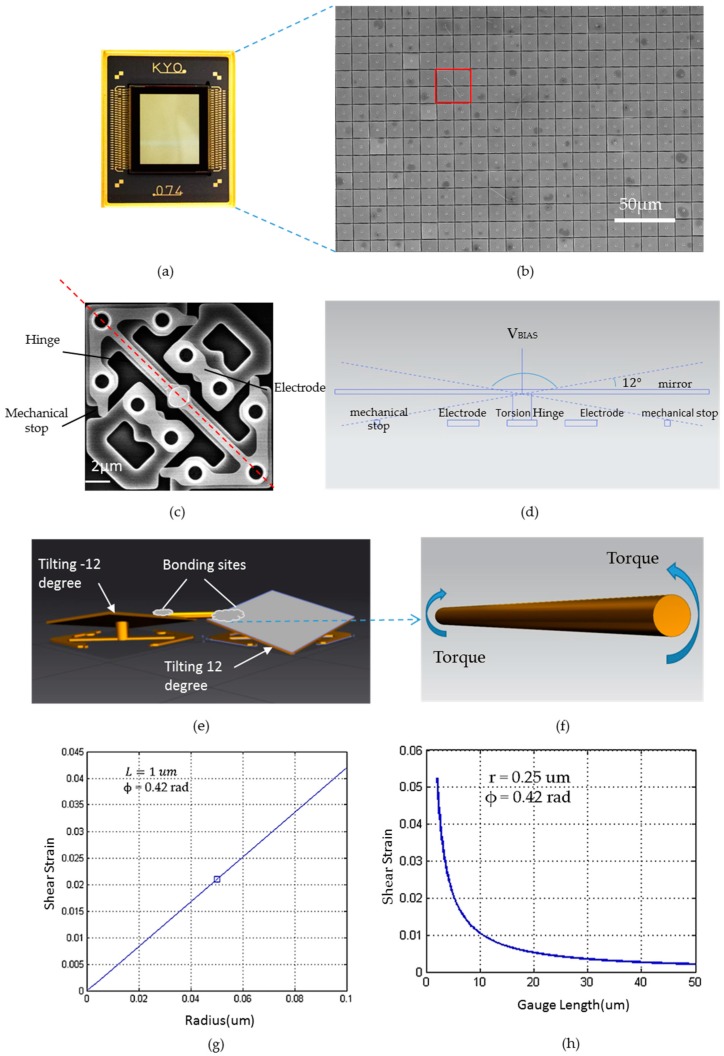
Scheme of the DMD-based torsional testing micromachine: (**a**) shows the DMD chip after removing the protection cover; (**b**) SEM image shows that millions of micromirror arrays are available on a single DMD chip, with one single nanowire (in red square) being clamped between two diagonally opposite micromirrors; (**c**) shows the micro-fabricated structure under a single micromirror, whose main components were torsion hinge and electrodes. Side view (**d**) shows how the electrostatic force between the mirror and the underneath electrodes actuate the micromirror. The schematic illustration (**e**) shows how two diagonal micromirrors tilting along the hinge can introduce the torsional straining on the nanowire clamped in-between (**f**). Graph (**g**) and (**h**) illustrate the relationship between shear strain and radius or gauge length.

Our platform was based on a customized Digital Light Processing (DLP) development kit from Texas Instrument, which was originally developed for programming and controlling the operation of DMD for various advanced optical/display applications [25]. Inside the DMD is a micro-electro-mechanical system (MEMS) consisting of hundreds of thousands of over one million of tiny switchable micro-mirrors with two stable mirror states (for example, +12° and −12° for most DMD devices) as shown in Figure 1b [26]. In the present work, the 0.7’’ 1024 × 768 Extended Graphics Array (XGA) TI DMD was used, in which the micromirrors were attached to the underlying torsion hinges as shown in Figure 1c. Under the effects of electrode potential, the mirror could be electrostatically attracted to address electrodes with the largest electrostatic field potential when bias/reset voltage was applied on it [27]. The mirror rotated until it came to rest against mechanical stops which limits the mirror rotation angle to +12° or −12°. Due to these characteristics, the rotation direction of individual micromirrors was fully controllable through input programming. 

For the purpose of mechanical loading, the tilting movement of the micromirrors in DMD could be used in applying torsional load on the individual 1D nanostructures, such as nanowires with diameters ~100–500 nm (or even larger) if they were positioned properly. Specifically, torsional deformation of nanowires/nanotubes could be realized by aligning and clamping the sample along the tilting (hinge) axis of the micromirrors (as shown in Figure 1e, which showed the corresponding out-of-plane movement). The maximum twisting angle of the sample could be totally 24°, when two mirrors were fully tilted toward the opposite directions (+12° for one mirror and −12° for another mirror). The maximum torsional strain can be then calculated as torsion strain γ = *r*·ϕ/*L*, where *r* is the sample radius, ϕ is the total torsion radian, *L* is gauge length of the sample (the actual strain depends on the true loading state when the nanowire samples were deformed). From Figure 1g,h, it is easy to find that the shear strain of nanowire is proportional to its radius and inversely proportional to its gauge length.

**Figure 2:**
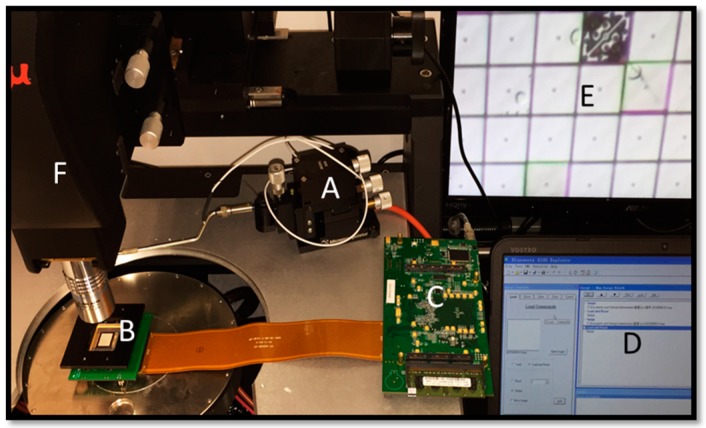
Experimental setup under an optical microscope: The micromanipulator (**A**) was used to bond nanowire onto the micromirrors of DMD chip (**B**), which connected with the digital light processing (DLP) circuit board (**C**) via data cable. In order to control the individual DMD pixel (micromirror), the customized programming software (**D**) will transmit the binary signal to (**C**). During the testing, the real time images (**E**) will be captured by a high magnification optical microscope (**F**) equipped with a high-speed camera.

Figure 2 shows the entire micromachine platform and their respective arrangement for testing under an optical microscope. After removing the kovar metal frame from the DMD, the nanowire specimen would be clamped onto the micromirrors with epoxy by a high-precision nanomanipulator. In order to achieve pure torsion, the axis of the nanowire should be well aligned along with the torsion hinge precisely. The bonding of the nanowire with the micromirror should be firm (each glue spot size about 2 μm) to avoid misalignment and sliding issues. The optical microscope which connected with a high-speed camera was used to capture the *in situ* image of the nanowire under loading. The state of each micromirror could be controlled by our customized software with Graphical User Interface (GUI) on the computer. During operation, the DLP circuit board controlling system decoded the programming information from the computer into the bit stream read by the DMD. The loading cycles and loading frequency could be input from the software easily. As the micromirror flipping frequency could be up to thousands of Hz, this platform is well suited for the desired high-cycle fatigue testing (and the DMD was proved to be reliable and stable after billions of operations [24]).

**Figure 3:**
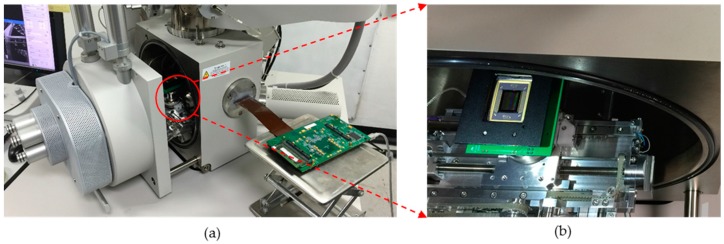
*In situ* SEM testing set-up: the digital light procession (DLP) circuit board in (**a**) is connected with the (**b**) DMD chip inside the SEM chamber through a data cable. The compact size of the DMD chip unit can ensure the remote operation via the data cable inside the SEM.

Most importantly, because of its small footprint and the cable-remote controlling mechanisms, this DMD-based testing platform can be ideal for the *in situ* scanning electron microscope (SEM) testing, allowing the real time observation of materials responses under torsion cyclic loading. This is one of the most attractive features of our platform as showed in Figure 3. With a customized SEM port for cable communication and remote control, we have successfully managed to put the DMD into a high resolution SEM (FEI Quanta 450 FEG SEM, Hillsboro, OR, USA) and made it work inside the SEM.

## 3. Platform Demonstration and Results

Based on this platform, cyclic torsional tests of nanowire samples with different diameters and gauge length have been demonstrated. The SEM images of the samples were always recorded before torsional fatigue tests. The effective torsion gauge length depended on the distance between the two glue clamping positions. The torsion test could be observed by the high-speed camera which connected to the microscope (optical or SEM). In order to make it easy to distinguish the three states of micromirrors according to brightness, we tilted the DMD a little bit with respect to the projecting light. It could be defined that if the micromirror became very bright, it meant it was on flat/ground state, which could reflect more light into the microscope. If the micromirror became very dark, it meant the micromirror flipped to “off” state or its angle was −12°, which reflected much of the light off the microscope. If the micromirror was gray (in between the two above states), it meant the micromirror flipped to “on” state or its angle was 12°, which reflected some of the light into the microscope but less than that of the flat state.

### 3.1. Demonstration of the Cyclic Loading with Different Loading Amplitudes

**Figure 4:**
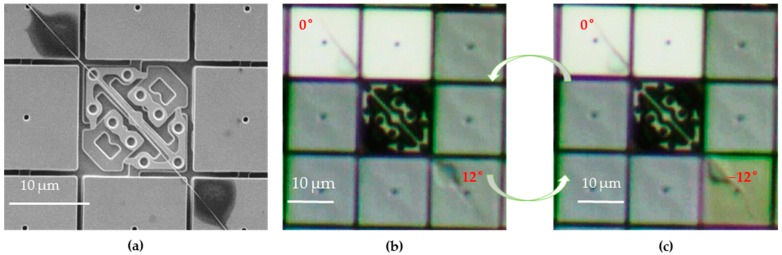
Cyclic torsional loading on a nanowire with twisting angle of 12°: (**a**) shows the SEM image of the 100 nm nanowire sample after the test. The lower right mirror flipped to 12° in (**b**) then quickly flipped to −12° in (**c**). Repeating the processes of (**b**) and (**c**) alternatively will induce the cyclic torsion loading on the nanowire sample.

We firstly demonstrated the torsional twisting of a silver nanowire with diameter ~100 nm and gauge length ~25 μm by 12° (see also the Supplementary Video S1). In this case, only one micromirror needed to flip, while another one was fixed all the way (at ground state as shown in Figure 4). During the test, the micromirror at the upper left corner was fixed, while another micromirror at the lower right firstly flipped to positive 12° (Figure 4b), and then quickly flipped to negative 12° (Figure 4c). As a result, repeating processes of (b) and (c) alternatively will induce the torsion loading on the nanowire. Under this loading pattern, the frequency could be up to 50 Hz. After more than one million loading cycles (8 h × 50 Hz) with 0.04% shear strain, the nanowire still did not break.

**Figure 5:**
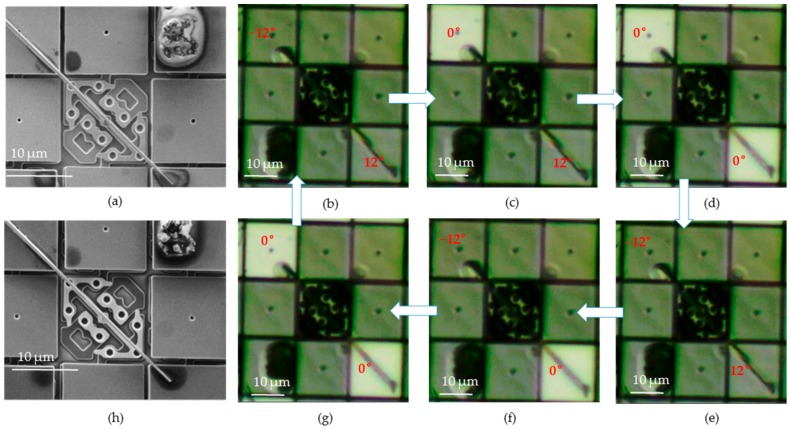
Cyclic torsional loading on a nanowire with maximum 24° twisting: From (**b**) to (**g**), the rotation angle changes as the picture shows. The maximum total torsion angle is 24° in this case (12° + 12°), with two diagonal mirrors titled in opposite directions. (**a**) shows the original nanowire with diameter ~500 nm before the test. (**h**) shows the same nanowire after the test.

To further demonstrate that this micromachine could be versatile for various 1D nanomaterials with different sizes, another nanowire sample with diameter ~500 nm and gauge length ~25 μm was also tested, but the torsion angle was 24° this time (see also the Supplementary Video S2). Figure 5 showed how the nanowire with diameter of 500 nm was twisted by DMD micromirrors during a full loading cycle as we designed (the “on” state is brighter than “off” state in this figure). The nanowire was very straight and boned precisely along the axis of hinge before the experiment as Figure 5a shows. In Figure 5b, which was named as the loading process, the micromirror at the upper left corner flipped to negative 12°, the micromirror at the lower right corner flipped to positive 12°. The nanowire would be twisted by 24° in total, which was the maximum twisting angle our platform can provide (fairly enough for most torsional fatigue studies). In Figure 5c, the upper one returned to flat state, and the lower one stayed at the negative 12° position. Then, in Figure 5d, both of the two micromirrors became flat. Figure 5c,d could be called the unloading process, in which the nanowire would become stress free. In Figure 5e, the loading process started again and both of them would quickly flip to −12° and 12°, respectively, at the same time. However, the unloading process in Figure 5f became different. The upper one remained statically at negative 12° position, but the lower one flipped back to flat state. At last, the upper one flipped back to flat as shown in Figure 5g. They would always repeat this loading pattern, as long as the DMD was running, to maintain the fatigue straining process. Because this loading pattern included more steps than that of Figure 4, its frequency was slightly lower and was about 20 Hz in this case. In addition, after almost 576,000 loading cycles (8 h × 20 Hz) with shear strain about 0.42%, the nanowire still stayed original as Figure 5h shows, indicating good fatigue resistance.

### 3.2. Demonstration of the Fatigue Failures of Nanowires under Cyclic Torsional Loading

**Figure 6:**
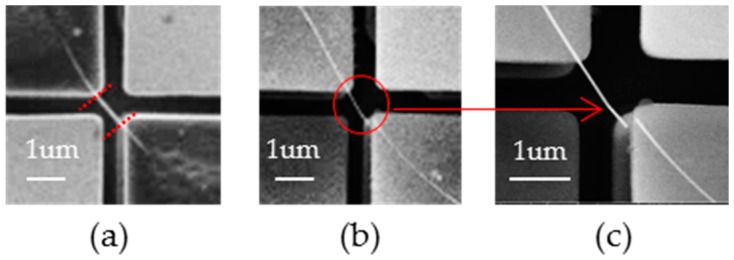
A Ag Nanowire with ~100 nm diameter has fractured after cyclic torsional loading: (**a**) shows the nanowire before the test. (**b**) shows the sample right after torsional fatigue failure after ~432,000 cycles. Note that the difference in contrast for the bonding points in (**a**) and (**b**) was due to the different electron beam voltages we used for SEM imaging the sample. We intentionally used low e-beam voltage (2 kV) for quantifying the boundary of clamping points in (**a**), while the bonding points appeared more “transparent” under high electron beam voltage (10 kV) in (**b**) and (**c**). (**c**) shows the close-up view of the fractured nanowire after stopped the test.

In order to increase the relative loading amplitude (e.g., shear strain) and see if the nanowire sample can be indeed fractured during torsional fatigue tests, the gauge length of the 100 nm diameter nanowire was decreased to about 1 μm. Figure 6 shows the nanowire before and after cyclic loading testing, and the experiment was done under optical microscope. The twisting pattern was similar as Figure 5. However, the nanowire fractured after about 432,000 (6 h × 20 Hz) of loading cycles with shear strain about 2.1%. We examine the sample before and after the test by SEM imaging and confirmed the fatigue failure of the nanowire sample with torsional fatigue.

**Figure 7:**
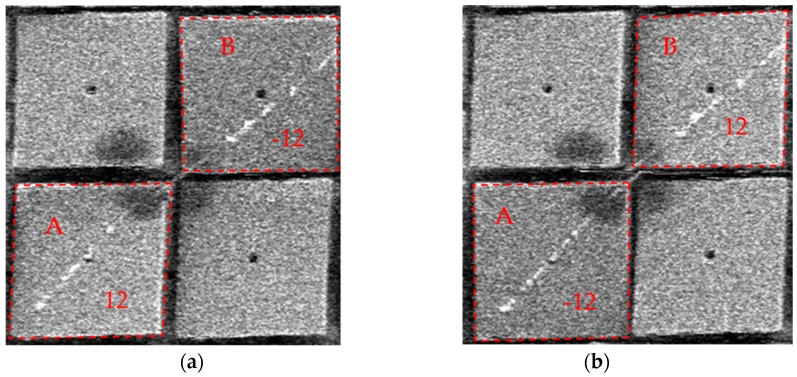
*In situ* SEM torsional fatigue testing of a 100 nm Ag nanowire: the two mirrors always tilted towards the opposite directions in each cycle. In (**a**) mirror A flipped to 12° and mirror B flipped to −12°. After almost four hours of cyclic loading, the nanowire fractured like (**b**).

Similarly, *in situ* SEM torsion fatigue tests were also demonstrated successfully, despite the dynamic *in situ* SEM imaging quality being low (in order to maintain the frame/scanning rate). Figure 7a,b (see also the Supplementary Video S3) show the corresponding *in situ* torsion loading of a Ag nanowire in SEM. Except for the loading pattern, the diameter of the nanowire (100 nm), the gauge length (1 μm) and torsion radian (24°) were all similar to Figure 6. The loading frequency, which was about 30 Hz, was faster than that of Figure 6. With the help of *in situ* SEM, the number of loading cycles before fracture became easier to obtain. The moment of fracture, as Figure 7b shows, was captured during the torsion test, after about 378,000 (3.5 h × 30 Hz) loading cycles with shear strain at about 2.1%. In addition, due to there being millions of mirrors on a single chip and them being able to be operated individually and independently, we could potentially test many samples at the same time, by clamping and testing them on different micro mirrors on the chip, which could speed up the whole fatigue study process in the future.

### 3.3. Quantifying the Strain Distribution along the Nanowire under Torsion

**Figure 8:**
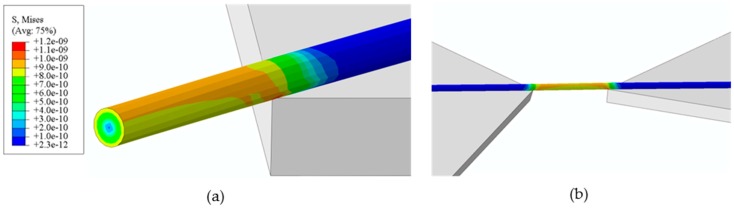
FEM analysis of the nanowire under torsional loading: The von Mises stress (also the strain) is relatively uniform along the gauge section of the nanowire sample in the elastic region but shows a gradient distribution in the cross-section toward the center (**a**). (**b**) shows the side view of the stress distribution in the whole nanowire specimen being twisted by total 24°.

Lastly, to quantify the strain distribution in the sample during torsion straining, finite element method (FEM) modeling is needed. Here, we used commercial software (ABAQUS) to simulate the nanowire torsional deformation actuated by the DMD tilting movements. One set of the simulation results was shown in Figure 8, indicating that the nanowires experienced relatively uniform deformation under pure torsional loading along their length direction. For the three kinds of demonstrated nanowire torsional testing experiments, the estimated max shear strain was ~0.04% for 100 nm silver nanowire (gauge length about 25 μm and torsion radian of 0.21 rad), ~0.42% for the 500 nm nanowires (gauge length about 25 μm and torsion radian of 0.42 rad), and ~2.1% for the 100 nm nanowires (gauge length about 1 μm and torsion radian of 0.42 rad), respectively. The shear strain of nanowire in Figure 3 was the same as that of Figure 7. In the future, the shear strain could be further elevated by adjusting the gauge length of the nanowire samples between the clamping points during the bonding process and/or by increasing the diameter of the samples (see also the Supplementary Video S4). It could be also seen that the maximum von Mises stress occurred at the edge of the nanowire and the lowest at the center of it, agreeing well with our expectation and solid mechanics theory. In the long run, assisted by real time SEM imaging, the FEM analysis will be beneficial for precisely quantifying the fatigue strain limit of the nanomaterial samples and for in-depth investigating of their failure mechanisms during the torsional loading. 

In the future, the frequency of the torsion loading by our setup can be further elevated by upgrading the firmware of the DLP controlling unit and the buffer memory of the DMD chip, which can, equivalently, further reduce the testing time, a major difficulty for many high cycle fatigue studies, and facilitate the desired *in situ* SEM testing. In addition, after trimming the corner of the micromirrors (such as those by focused ion beam (FIB)) to make their edge flat, this micromachine could be even used to test 2D nanomaterials, such as graphene or MoS_2_ multiplayers, for their cyclic deformation property and stability. In such cases, high resolution electron microscopy will definitely be needed, while our platform has the exact strength for such *in situ* SEM testing.

## 4. Conclusions

In summary, a novel torsional fatigue testing micromachine for various 1D nanostructures based on a “digital micromirror device” (DMD) has been successfully developed, and we demonstrated its feasibility by characterizing the high-cycle fatigue behavior of several nanowires with different diameters and shear strains. In addition, its small footprint enables the desired *in situ* SEM testing, which is not only helpful in monitoring the fatigue testing status but also in providing critical insights on the deformation processes and underlying mechanisms governing the nanomaterials’ fatigue behavior. The successful adoption of such a well-established MEMS product (DMD) for the challenging nano-fatigue testing would likely be a cost-efficient yet robust solution with high performance and reliability. This micromachine solution could also inspire the design and development of novel MEMS-based micromechanical testing systems for other kinds of nanoscale materials.

## Supplementary Materials

The following are available online at http://www.mdpi.com/2072-666X/7/3/49/s1. Video S1: The 100 nm nanowire under 12° torsion testing; Video S2: The 500 nm nanowire under 24° torsion testing; Video S3: *in situ* SEM nanowire torsion testing; Video S4: *in situ* SEM torsion testing with a larger-diameter sample.

## References

[1] McAlpine M.C., Ahmad H., Wang D. (2007). Highly ordered nanowire arrays on plastic substrates for ultrasensitive flexible chemical sensors. Nat. Mater..

[2] Nair P.R., Alam M. (2007). Design considerations of silicon nanowire biosensors. IEEE Trans. Electron Devices.

[3] Fan Z., Ho J.C., Takahashi T., Yerushalmi R., Takei K., Ford A.C., Chueh Y.L., Javey A. (2009). Toward the development of printable nanowire electronics and sensors. Adv. Mater..

[4] Gudiksen M.S., Lauhon L.J., Wang J., Smith D.C., Lieber C.M. (2002). Growth of nanowire superlattice structures for nanoscale photonics and electronics. Nature.

[5] Yao J., Yan H., Das S., Klemic J.F., Ellenbogen J.C., Lieber C.M. (2014). Nanowire nanocomputer as a finite-state machine. Proc. Natl. Acad. Sci. USA.

[6] Uchic M.D., Dimiduk D.M., Florando J.N., Nix W.D. (2004). Sample dimensions influence strength and crystal plasticity. Science.

[7] Chang P.C., Chien C.J., Stichtenoth D., Ronning C., Lu J.G. (2007). Finite size effect in ZnO nanowires. Appl. Phys. Lett..

[8] Wu B., Heidelberg A., Boland J.J. (2005). Mechanical properties of ultrahigh-strength gold nanowires. Nat. Mater..

[9] Zhu T., Li J., Ogata S., Yip S. (2009). Mechanics of ultra-strength materials. MRS Bull..

[10] Lu Y., Lou J. (2011). Quantitative in-situ nanomechanical characterization of metallic nanowires. JOM.

[11] Gianola D.S., Eberl C. (2009). Micro-and nanoscale tensile testing of materials. JOM.

[12] Boyce B., Huang J., Miller D., Kennedy M. (2010). Deformation and failure of small-scale structures. JOM.

[13] Namazu T., Isono Y. High-cycle fatigue test of nanoscale Si and SiO_2_ wires based on AFM technique. Proceedings of the 16th IEEE Annual International Conference on Micro Electro Mechanical Systems (MEMS 2003).

[14] Gao Z., Ding Y., Lin S., Hao Y., Wang Z.L. (2009). Dynamic fatigue studies of ZnO nanowires by in-situ transmission electron microscopy. Phys. Status Solidi RRL..

[15] Li P., Liao Q., Yang S., Bai X., Huang Y., Yan X.Q., Zhang Z., Liu S., Lin P., Kang Z. (2014). *In situ* transmission electron microscopy investigation on fatigue behavior of single ZnO wires under high-cycle strain. Nano Lett..

[16] Ensslen C., Kraft O., Mönig R., Xu J., Zhang G.P., Schneider R. (2014). Mechanical annealing of Cu–Si nanowires during high-cycle fatigue. MRS Commun..

[17] Hosseinian E., Pierron O.N. (2013). Quantitative *in situ* TEM tensile fatigue testing on nanocrystalline metallic ultrathin films. Nanoscale.

[18] Kumar S., Alam M.T., Haque M.A. (2011). Fatigue Insensitivity of Nanoscale Freestanding Aluminum Films. J. Microelectromech. Syst..

[19] Lu Y., Ganesan Y., Lou J. (2010). A multi-step method for *in situ* mechanical characterization of 1-D nanostructures using a novel micromechanical device. Exp. Mech..

[20] Haque M.A., Espinosa H.D., Lee H.J. (2010). MEMS for *in situ* testing—Handling, actuation, loading, and displacement measurements. MRS Bull..

[21] Pant B., Allen B.L., Zhu T., Gall K., Pierron O.N. (2011). A versatile microelectromechanical system for nanomechanical testing. Appl. Phys. Lett..

[22] Saini K., Kumar N. (2014). Torsional deformation behavior of cracked gold nano-wires. Acta Mech..

[23] Jiang S., Zhang H., Zheng Y., Chen Z. (2009). Atomistic study of the mechanical response of copper nanowires under torsion. J. Phys. D Appl. Phys..

[24] Gao Y., Wang F., Zhu T., Zhao J. (2010). Investigation on the mechanical behaviors of copper nanowires under torsion. Comput. Mater. Sci..

[25] Hornbeck L.J. (1997). Digital light processing for high-brightness high-resolution applications. Electronic Imaging'97.

[26] Douglass M.R. Lifetime estimates and unique failure mechanisms of the digital micromirror device (DMD). Proceedings of the 36th Annual IEEE International Reliability Physics Symposium.

[27] Dudley D., Duncan W.M., Slaughter J. (2003). Emerging digital micromirror device (DMD) applications. Micromachining and Microfabrication.

